# Distribution of allele frequencies at *TTN g.231054C > T*, *RPL27A g.3109537C > T *and AKIRIN2 c.*188G > A between Japanese Black and four other cattle breeds with differing historical selection for marbling

**DOI:** 10.1186/1756-0500-4-10

**Published:** 2011-01-20

**Authors:** Naoto Watanabe, Youichi Satoh, Tatsuo Fujita, Takeshi Ohta, Hiroyuki Kose, Youji Muramatsu, Takuji Yamamoto, Takahisa Yamada

**Affiliations:** 1 Oita Prefectural Institute of Animal Industry, Takeda, Oita 878-0201, Japan; 2 Iwate Agricultural Research Center Animal Industry Research Institute, Takizawa, Iwate 020-0173, Japan; 3 Laboratory of Animal Genetics, Graduate School of Science and Technology, Niigata University, Nishi-ku, Niigata 950-2181, Japan; 4 Department of Life Science, Division of Natural Sciences, International Christian University, Mitaka, Tokyo 181-8585, Japan; 5 Department of Nutritional Sciences for Well-being, Faculty of Health Sciences for Welfare, Kansai University of Welfare Sciences, Kashiwara, Osaka 582-0026, Japan

## Abstract

**Background:**

Marbling defined by the amount and distribution of intramuscular fat, so-called *Shimofuri*, is an economically important trait of beef cattle in Japan. Our previous study detected 3 single nucleotide polymorphisms (SNPs), *g.231054C > T*, *g.3109537C > T *and c.*188G > A, respectively, in the 5' flanking region of the *titin *(*TTN*), the 5' flanking region of the *ribosomal protein L27a *(*RPL27A*) and the 3' untranslated region of the *akirin 2 *genes (*AKIRIN2*), which have been considered as positional functional candidates for the genes responsible for marbling, and showed association of these SNPs with marbling in Japanese Black beef cattle. In the present study, we investigated the allele frequency distribution of the 3 SNPs among the 5 cattle breeds, Japanese Black, Japanese Brown, Japanese Shorthorn, Holstein and Brown Swiss breeds.

**Findings:**

We genotyped the *TTN g.231054C > T*, *RPL27A g.3109537C > T *and AKIRIN2 c.*188G > A SNPs by polymerase chain reaction-restriction fragment length polymorphism method, using 101 sires and 1,705 paternal half sib progeny steers from 8 sires for Japanese Black, 86 sires and 27 paternal half sib progeny steers from 3 sires for Japanese Brown, 79 sires and 264 paternal half sib progeny steers from 14 sires for Japanese Shorthorn, 119 unrelated cows for Holstein, and 118 unrelated cows for Brown Swiss breeds. As compared to the frequencies of the *g.231054C > T T*, *g.3109537C > T T *and c.*188G > A A alleles, associated with high marbling, in Japanese Black breed that has been subjected to a strong selection for high marbling, those in the breeds, Japanese Shorthorn, Holstein and Brown Swiss breeds, that have not been selected for high marbling were null or lower. The Japanese Brown breed selected slightly for high marbling showed lower frequency than Japanese Black breed in the *g.3109537C > T T *allele, whereas no differences were detected between the 2 breeds in the frequencies of the *g.231054C > T T *and c.*188G > A A alleles.

**Conclusions:**

Based on this finding, we hypothesized that the pressure of the strong selection for high marbling in Japanese Black breed has increased the frequencies of the *T*, *T *and *A *alleles at the *TTN g.231054C > T*, *RPL27A g.3109537C > T *and AKIRIN2 c.*188G > A SNPs, respectively. This study, together with the previous association studies, suggested that the 3 SNPs may be useful for effective marker-assisted selection to increase the levels of marbling.

## Findings

### Background

Marbling characterized by the amount and distribution of intramuscular fat, of which the increase improves the palatability and acceptability of the meat [1-3], is an economically important trait of beef cattle in Japan [4].

The *titin *(*TTN*) [5], *ribosomal protein L27a *(*RPL27A*) [6] and *akirin 2 *(*AKIRIN2*) genes [7] have been regarded as positional functional candidates for the genes responsible for marbling. We have reported that 3 single nucleotide polymorphisms (SNPs), referred to as *g.231054C > T*, *g.3109537C > T *and c.*188G > A, respectively, were detected in the 5' flanking region of the *TTN *[5], the 5' flanking region of the *RPL27A *[6] and the 3' untranslated region of the *AKIRIN2 *[7], between low-marbled and high-marbled steer groups, which were shown to have *TTN*, *RPL27A *and *AKIRIN2 *expression differences in *musculus longissimus *muscle [8]. Further, we have shown association of the 3 SNPs with marbling in Japanese Black beef cattle, with the *g.231054C > T T *[5], *g.3109537C > T T *[6] and c.*188G > A A alleles [7] resulting in higher levels of marbling.

There has been a strong selection for high marbling in Japanese Black breed and a slight selection in Japanese Brown breed, but not in other breeds such as Japanese Shorthorn, Holstein, and Brown Swiss, over the past 50 years [9-11]. In the present study, we investigated the allele frequency distribution of the 3 SNPs among the 5 cattle breeds.

### Samples and SNP genotyping

We used 101 sires (from 43 fathers, 1-6 sires per father), 86 sires (from 41 fathers, 1-5 sires per father), 79 sires (from 50 fathers, 1-3 sires per father), 119 cows (from 119 independent fathers) and 118 cows (from 118 independent fathers), respectively, for Japanese Black, Japanese Brown, Japanese Shorthorn, Holstein and Brown Swiss breeds. There was no strong bias for a specific father or a specific maternal grandfather of the sires or cows within each breed, and the animal panel for each breed likely represents a random sample of population of each breed. Furthermore, we used 1,705 (from 8 sires, 61-521 steers per sire), 27 (from 3 sires, 8-10 steers per sire) and 264 paternal half-sib progeny steers (from 14 sires, 17-33 steers per sire), respectively, for Japanese Black, Japanese Brown and Japanese Shorthorn breeds. The dams of these progeny steers within each breed were considered to be a random mating population of each breed.

Semen, blood or adipose tissues were collected from these animals for SNP genotyping. These materials were sampled by the Oita Prefectural Institute of Animal Industry (Oita, Japan) for Japanese Black, Holstein and Brown Swiss breeds, the Kumamoto Prefectural Agricultural Research Center Institute of Animal Industry (Kumamoto, Japan) for Japanese Brown breed and the Iwate Agricultural Research Center Animal Industry Research Institute (Iwate, Japan) for Japanese Shorthorn breed. DNA was prepared from the materials according to standard protocols. This study conformed to the guidelines for animal experimentation of the Graduate School of Science and Technology, Niigata University (Niigata, Japan).

The *g.231054C > T*, *g.3109537C > T *and c.*188G > A SNPs were genotyped by polymerase chain reaction-restriction fragment length polymorphism method using restriction enzymes *Hpy*CH4III [5], *Bsu*36I [6] and *Fok*I [7], respectively.

The *g.231054C > T* , *g.3109537C > T* and c.*188G > A have been submitted to the dbSNP with ss accession number 283947863, 275523807 and 283947865, respectively.

### Allele frequency distribution

Table 1 summarizes the allele frequency distribution of the *g.231054C > T*, *g.3109537C > T *and c.*188G > A SNPs among the 5 cattle breeds, Japanese Black, Japanese Brown, Japanese Shorthorn, Holstein and Brown Swiss breeds. Departures from the Hardy-Weinberg equilibrium were tested for each of the 3 SNPs in each of sire or cow populations of the 5 cattle breeds, except for the *g.231054C > T* in Holstein and Brown Swiss breeds in which the *C* allele was fixed. In case of the test for the c.*188G > A in Japanese Shorthorn breed, in which 76 animals were homozygous for the *G* allele, and 2 animals heterozygous for the *G* and *A* alleles and 1 animal homozygous for the *A* allele, the 2 heterozygotes and the 1 *AA* homozygote were merged. Statistically significant departures at the 5% level were not observed for all the tests for the 3 SNPs in the 5 cattle breeds. In each of Japanese Black, Japanese Brown and Japanese Shorthorn breeds, for each of the 3 SNPs, statistical comparisons between the allele frequencies in sires and the data estimated by inferred maternal alleles in half-sib progeny steers were performed by chi-square test or Fisher's exact probability test. No statistically significant differences were detected between the 2 allele frequencies for each of the 3 SNPs in each of the 3 breeds (Table 2).

**Table 1:** Distribution of allele frequencies at the *g.231054C > T*, *g.3109537C > T *and c.*188G > A among the 5 cattle breeds

	Frequency					
	
	*g.231054C > T*		*g.3109537C > T*		c.*188G > A	
	
Breed	*C *allele	*T *allele	*C *allele	*T *allele	*G *allele	*A *allele
Japanese Black-sires	0.728	0.272	0.589	0.411	0.500	0.500
Japanese Black-progeny steers	0.687	0.313	0.583	0.417	0.476	0.524
Japanese Brown-sires	0.724	0.276	0.732	0.268	0.430	0.570
Japanese Brown-progeny steers	0.759	0.241	0.759	0.241	0.389	0.611
Japanese Shorthorn-sires	0.910	0.090	0.702	0.298	0.975	0.025
Japanese Shorthorn-progeny steers	0.894	0.106	0.693	0.307	0.981	0.019
Holstein	1.000	0.000	0.695	0.305	0.790	0.210
Brown Swiss	1.000	0.000	0.763	0.237	0.787	0.213

**Table 2:** Statistical significance for differences in allele frequencies for each of the *g.231054C > T*, *g.3109537C > T *and c.*188G > A between the 5 cattle breeds

	Breed^†^						
	
	JB-progeny	JBR-sires	JBR-progeny	JSH-sires	JSH-progeny	HOL	BS
*g.231054C > T*							
JB-sires	n.s.	n.s.	n.s.	**	**	**	**
JB-progeny		n.s.	n.s.	**	**	**	**
JBR-sires			n.s.	**	**	**	**
JBR-progeny				*	*	**	**
JSH-sires					n.s.	**	**
JSH-progeny						**	**
HOL							n.s.
*g.3109537C > T*							
JB-sires	n.s.	**	n.s.	*	*	*	**
JB-progeny		**	n.s.	**	**	**	**
JBR-sires			n.s.	n.s.	n.s.	n.s.	n.s.
JBR-progeny				n.s.	n.s.	n.s.	n.s.
JSH-sires					n.s.	n.s.	n.s.
JSH-progeny						n.s.	n.s.
HOL							n.s.
c.*188G > A							
JB-sires	n.s.	n.s.	n.s.	**	**	**	**
JB-progeny		n.s.	n.s.	**	**	**	**
JBR-sires			n.s.	**	**	**	**
JBR-progeny				**	**	**	**
JSH-sires					n.s.	**	**
JSH-progeny						**	**
HOL							n.s.

n.s.: not significant.

*P < 0.05.

**P < 0.01.

^†^JB, Japanese Black; JBR, Japanese Brown; JSH, Japanese Shorthorn; HOL, Holstein; BS, Brown Swiss.

Further, statistical comparisons for the allele frequencies between breeds were performed. Statistically significant differences were detected between Japanese Black or Japanese Brown breeds and the other breeds for the *g.231054C > T*, between Japanese Black breed and the other breeds including Japanese Brown (for sires) for the *g.3109537C > T *and between Japanese Black or Japanese Brown breeds and the other breeds for the c.*188G > A (Table 2). We have previously reported that the *g.231054C > T T*, *g.3109537C > T T *and c.*188G > A A alleles are associated with high marbling [5-7]. The frequencies of these alleles in Japanese Black breed, which has been subjected to a strong selection for high marbling, were 0.272 in the sires and 0.313 in the progeny steers for the *g.231054C > T T*, 0.411 in the sires and 0.417 in the progeny steers for the *g.3109537C > T T *and 0.500 in the sires and 0.524 in the progeny steers for the c.*188G > A A alleles (Table 1). As expected, as compared to these frequencies in Japanese Black breed, those in the breeds that have not been selected for high marbling were null (Holstein and Brown Swiss for the *g.231054C > T T*) or lower (Japanese Shorthorn for the *g.231054C > T T*, Japanese Shorthorn, Holstein and Brown Swiss for the *g.3109537C > T T *and Japanese Shorthorn, Holstein and Brown Swiss for the c.*188G > A A) (Tables 1 and 2). Additionally, the Japanese Brown breed which has been slightly selected for high marbling, showed significantly lower frequency (for sires) than Japanese Black breed and indistinguishable frequency from Japanese Shorthorn, Holstein and Brown Swiss breeds in the *g.3109537C > T T *allele, whereas no significant differences were detected between Japanese Black and Japanese Brown breeds in the *g.231054C > T T *and c.*188G > A A alleles and the frequencies in Japanese Brown breed were significantly higher than those in Japanese Shorthorn, Holstein and Brown Swiss breeds (Tables 1 and 2). There were no significant differences between Holstein and Brown Swiss breeds. We should note that all the *P* values reported in this study were nominal, with no correction for multiple testing.

This finding leads to the hypothesis that the pressure of the strong selection and the slight selection for high marbling in Japanese Black and in Japanese Brown breeds, respectively, has increased the frequencies of the *TTN g.231054C > T T*, *RPL27A g.3109537C > T T *and AKIRIN2 c.*188G > A A alleles and of the *TTN g.231054C > T T *and AKIRIN2 c.*188G > A A alleles, assuming that the 3 alleles have experienced a selective sweep. Thus, this study, together with the previous association studies [5-7], suggests that the 3 SNPs may be useful for effective marker-assisted selection to increase the levels of marbling. In previous study, we supposed that the *TTN g.231054C > T *[5], *RPL27A g.3109537C > T *[6] and AKIRIN2 c.*188G > A SNPs [7] might affect gene expression and also marbling through affecting promoter activity or mRNA stability. The high frequencies of the *g.231054C > T T*, *g.3109537C > T T *and c.*188G > A A alleles and of the *g.231054C > T T *and c.*188G > A A alleles in Japanese Black and Japanese Brown breeds, respectively, out of the 5 cattle breeds may imply that the 3 SNPs have an direct impact on marbling. On the other hand, the degree of genetic influence of European breeds on Japanese Brown breed [11] and Japanese Shorthorn breed [12,13] is considered to be high to some extent, although both of these, and the Japanese Black breed, are generically known as Wagyu cattle. Thus, we cannot exclude the possibility that the difference in the allele frequency distribution of the *TTN g.231054C > T*, *RPL27A g.3109537C > T *and AKIRIN2 c.*188G > A SNPs among the 5 cattle breeds in this study is attributable to the genetic influence of European breeds rather than the influence of selection for high marbling. Additionally, Japanese Black and Japanese Brown breeds are known to have a small effective population size. Thus, we cannot exclude the possibility that genetic drift could explain the difference in the allele frequency distribution of the 3 SNPs among breeds. Further investigation of the allele frequency distribution of the *TTN g.231054C > T*, *RPL27A g.3109537C > T *and AKIRIN2 c.*188G > A SNPs using Asian indigenous cattle, extremely high-marbled and extremely low-marbled cattle groups within each breed or historical samples of either tissue or semen from Wagyu cattle from 50 or more years ago will be needed to deny these possibilities.

## Competing interests

The authors declare that they have no competing interests.

## Authors' contributions

NW participated in the sample collection and carried out the SNP genotyping. YS and TF participated in the sample collection. TO, HK, and YM carried out the SNP genotyping. TY helped to draft the manuscript. TY participated in the design and coordination of the study and drafted the manuscript. All authors read and approved the final manuscript.
